# Psychometric profile of the Ages and Stages Questionnaires, Japanese translation

**DOI:** 10.1111/ped.13990

**Published:** 2019-11-26

**Authors:** Hidetoshi Mezawa, Sayaka Aoki, Shoji F. Nakayama, Hiroshi Nitta, Natsuha Ikeda, Keiko Kato, Satoshi Tamai, Makoto Takekoh, Masafumi Sanefuji, Shouichi Ohga, Masako Oda, Hiroshi Mitsubuchi, Ayako Senju, Koichi Kusuhara, Mari Kuwajima, Tatsuya Koeda, Yukihiro Ohya, Keiji Hashimoto

**Affiliations:** ^1^ Developmental Evaluation Center National Center for Child Health and Development Tokyo Japan; ^2^ Allergy Center National Center for Child Health and Development Tokyo Japan; ^3^ Japan Environment and Children's Study Programme Office National Institute for Environmental Studies Tsukuba Ibaraki Japan; ^4^ Regional Center for Pilot Study of Japan Environment and Children's Study Kyushu University Fukuoka Japan; ^5^ Regional Center for Pilot Study of Japan Environment and Children's Study Kumamoto University Kumamoto Japan; ^6^ Regional Center for Pilot Study of Japan Environment and Children's Study University of Occupational and Environmental Health Kitakyushu Japan; ^7^ Regional Center for Pilot Study of Japan Environment and Children's Study Jichi Medical University Shimotsuke Japan; ^8^ Department of Psychosocial Medicine National Center for Child Health and Development Tokyo Japan

**Keywords:** Ages and Stages Questionnaire, development, Japan Environment and Children's Study, screening tool, validation

## Abstract

**Background:**

This study assessed the psychometric profile of 10 questionnaires (every 6 months, from 6 to 60 months) from the Japanese translation of the Ages and Stages Questionnaires, third edition (J‐ASQ‐3).

**Methods:**

Data from 439 children in a birth cohort were used to identify the J‐ASQ‐3 score distribution, establish cut‐off scores, and calculate the instrument's internal consistency. Data were also collected from 491 outpatients to examine J‐ASQ‐3 test–retest reliability and concurrent validity, which was examined using the Kyoto Scale of Psychological Development (KSPD) and the Japanese version of the Denver Developmental Screening Test II (J‐Denver II). Both the original and the alternative screening criteria of the ASQ‐3 were used (failure in at least one and at least two domains, respectively).

**Results:**

Cronbach's alpha for each J‐ASQ‐3 subscale on each questionnaire ranged from 0.45 to 0.89. Test–retest reliability was >0.75 for the subscales on almost all questionnaires. Concurrent validity was also adequate. In comparison with the screening results of the KSPD, the overall sensitivity and specificity were 96.0% and 48.8%, respectively, when the ASQ‐3 original criterion was used, and 92.1% and 74.9%, respectively, when the alternative criterion was used. In comparison with the screening results of the J‐Denver II, the overall sensitivity and specificity were 75.6% and 74.7%, respectively, when the ASQ‐3 original criterion was used, and 56.3% and 93.0%, respectively, when the alternative criterion was used.

**Conclusions:**

This study quantified the psychometric profiles of the Japanese translations of 10 ASQ‐3 questionnaires. We demonstrated the validity of the J‐ASQ‐3 and determined new cut‐off scores. Further studies with larger samples from a greater range of locations are required to clarify the suitability of this tool for all Japanese children.

The Ages and Stages Questionnaires, third edition (ASQ‐3), is a screening tool for developmental delay used for children aged between 1 and 66 months.^1^ The tool captures developmental delay in five domains: communication, gross motor skills, fine motor skills, problem solving, and personal–social characteristics. The ASQ‐3 has been widely used in clinical and research settings in the USA because it is easy to use and has high reliability and validity. It has also been translated into several languages.^2^, ^3^, ^4^, ^5^

In Japan, approximately 10 different instruments are used to assess the development of preschool children, but none is appropriate for use in a large‐scale survey, the results of which are to be compared with those from other countries. For example, the Kyoto Scale for Psychological Development (KSPD) and the Tanaka–Binet Intelligence Test are widely used to assess young children's development in Japan, but they must be administered face to face.^6^, ^7^ The Japanese version of the Denver Developmental Screening Test II (J‐Denver II) is frequently used in Japan, but it does not identify the specific areas in which the child shows delay.^8^ In addition, the original English‐language version of the J‐Denver II is likely to overestimate the number of children who are developmentally delayed.^9^ The Kinder Infant Development Scale is a parent‐rated questionnaire that assesses development in several different domains, but only a Japanese version of the scale exists.^10^

The ASQ‐3 is an appropriate tool for a large‐scale survey. It is a parent‐rated questionnaire that takes only 10–15 min to complete. It assesses the child's development in five domains with high reliability and validity, and has been used frequently and internationally. At the time of the current study, however, there was no Japanese version of the ASQ‐3 available for use.

The purpose of the present study was to quantify the psychometric profile of the Japanese translation of the ASQ‐3 (J‐ASQ‐3). For this study, the translation was performed under contract with Brookes Publishing Company, which currently restricts use of the translated version to one specific study, the Japan Environment and Children's Study (JECS; for details, see Kawamoto *et al*.^11^ and Michikawa *et al*.^12^). We evaluated internal consistency and test–retest reliability, explored the score distribution and determined appropriate cut‐off scores, and examined the scale's specificity and sensitivity using the KSPD and J‐Denver II as reference tests.

## Methods

### Subjects and data collection

There were two groups of study participants: participants in the JECS pilot study and patients in two outpatient clinics: the National Center for Child Health and Development (NCCHD) and the Nico Children Clinic in Setagaya, Tokyo, Japan. Data from participants in the JECS pilot study were used to identify the J‐ASQ‐3 score distribution for healthy children and to calculate its internal consistency. Data from patients in the outpatient clinics were used to quantify the test–retest reliability and concurrent validity of the J‐ASQ‐3.

#### JECS pilot study participants

The JECS pilot study has been conducted in advance of the JECS main study in four locations in Japan in cooperation with four universities (Kyusyu University, University of Occupational and Environmental Health, Kumamoto University, and Jichi Medical University). The pilot study was approved by the Institutional Review Boards of the National Institute for Environmental Studies and each of the four universities (Kyusyu University, 20‐70; University of Occupational and Environmental Health, 08‐091; Kumamoto University, Epidemiology 59; Jichi Medical University, A09‐23; and National Institute for Environmental Studies, 2018‐002). The participants’ mothers were recruited between February 2009 and March 2010. The inclusion criteria were as follows: (i) pregnant; (ii) living in the designated study area and expected to reside continually in the area; (iii) able to understand the study procedures and fill out the questionnaires without support; and (iv) had signed the study informed consent form. Data were collected for 439 children (222 boys, 204 girls, and 13 unknowns). Of these children, 33 were born preterm (of these, only three were born before 32 gestational weeks) and 53 were born with low birthweight (of these, only three had birthweight <1,500 g). Based on their parent's responses during the survey, 11 children were diagnosed with autism, 12 with mental retardation, and one with epilepsy. These high‐risk conditions (preterm, low birthweight, autism, mental retardation, and epilepsy) sometimes co‐occurred with each other. In total, 84 children (19.1%) were considered to be in the high‐risk group for developmental difficulties. The demographic information is presented in Table S1.

The participants’ mothers were asked to complete the mailed questionnaires every 6 months after their children were born. The J‐ASQ‐3 was included as part of the survey questionnaire collected from age 6 to 60 months. The following responses were excluded: (i) those with missing items, and (ii) those collected when the child was older than the age range covered by the corresponding questionnaire. Some mothers responded to all the questionnaires, and others completed only one or a few. Therefore, the number of respondents who completed each of the questionnaires varied (Table 1). The total number of questionnaires completed during the entire pilot study varied among the participants (Table S2).

**Table 1:** Descriptive statistics and cut‐off scores for each J‐ASQ‐3 version and subscale (*n* = 439)

Questionnaire (months)	*n*	Mean	SD	Cut‐off	Monitoring	Mean diff. from ASQ	*n*	Mean	SD	Cut‐off	Monitoring	Mean diff. from ASQ
	Communication						Gross motor					
6	47	44.36*	10.71	22.93	33.65	−4.54	47	39.04*	11.96	15.12	27.08	−6.60
12	145	35.41*	15.44	4.53	19.97	−7.80	148	45.07*	17.82	9.43	27.25	−4.95
18	174	32.82*	13.50	5.82	19.32	−9.51	174	55.63*	9.02	37.59	46.61	0.26
24	291	46.41*	16.04	14.33	30.37	−4.71	294	54.59*	7.73	39.13	46.86	−0.26
30	319	51.99*	12.99	26.01	39.00	−1.82	321	54.60	8.12	38.36	46.48	1.06
36	317	53.15	11.60	29.95	41.55	1.27	323	55.9*	8.32	39.26	47.58	1.22
42	359	54.11*	10.09	33.94	44.02	4.09	362	56.44*	6.48	43.48	49.96	2.41
48	346	53.93	10.44	33.05	43.49	1.01	344	54.93*	8.58	37.78	46.35	2.22
54	347	55.86*	10.40	35.07	45.47	2.07	344	55.42*	9.09	37.23	46.33	1.44
60	250	49.18*	10.77	27.65	38.41	−3.24	276	51.70	10.12	31.47	41.59	−0.47
	Fine motor						Problem solving					
6	48	44.17*	13.97	16.24	30.20	−4.76	48	47.71	10.72	26.27	36.99	−2.70
12	149	47.35*	10.94	25.47	36.41	−4.91	146	42.77*	13.70	15.37	29.07	−6.34
18	178	49.24*	11.24	26.76	38.00	−3.35	174	42.67*	13.37	15.93	29.30	−3.34
24	289	48.7*	7.61	33.48	41.09	−2.96	290	50.1*	10.36	29.38	39.74	0.60
30	315	46.98	12.98	21.03	34.01	0.20	320	50.36	12.29	25.78	38.07	0.18
36	310	50.73*	11.41	27.91	39.32	3.66	289	52.06	11.02	30.03	41.04	0.09
42	355	53.31*	9.99	33.33	43.32	5.76	361	54.96*	9.39	36.18	45.57	3.42
48	342	51.94*	10.88	30.18	41.06	6.59	344	54.24*	10.19	33.87	44.06	1.46
54	336	49.18*	11.00	27.19	38.19	3.06	347	54.52*	9.67	35.19	44.86	3.27
60	247	50.28	12.86	24.56	37.42	−1.29	273	56.54*	8.58	39.39	47.96	3.95
	Personal−social											
6	48	37.19*	14.66	7.88	22.53	−11.12						
12	149	36.91*	15.98	4.95	20.93	−8.88						
18	175	45.17*	10.30	24.57	34.87	−2.59						
24	291	43.38*	9.08	25.22	34.30	−7.79						
30	319	50.2*	10.25	29.70	39.95	−1.67						
36	308	50.65*	10.38	29.89	40.27	−2.17						
42	364	54.66*	9.09	36.48	45.57	3.27						
48	335	53.25*	9.12	35.01	44.13	2.91						
54	324	55.31*	7.94	39.43	47.37	2.54						
60	276	56.63*	6.84	42.95	49.79	1.79						

* *P* < 0.05 (J‐ASQ‐3 mean score vs original ASQ‐3 score). ASQ‐3, Ages and Stages Questionnaires, third edition; J‐ASQ‐3, Japanese version of the Ages and Stages Questionnaires, third edition.

#### NCCHD and Nico Children's Clinic in Setagaya

**Figure 1:**
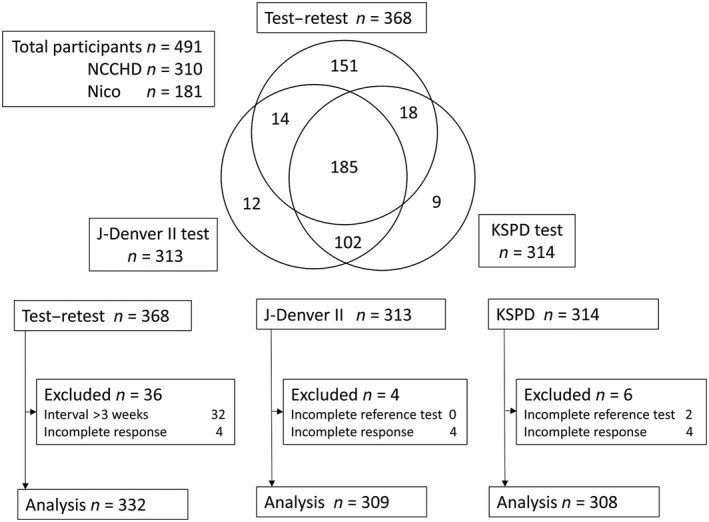
Venn diagram and flow charts of patients from the National Center for Child Health and Development (NCCHD) and the Nico Children Clinic in Setagaya. Incomplete response refers to participants with a response set containing three or more missing items in a domain. J‐Denver‐II, Japanese version of the Denver Developmental Screening Test; KSPD, Kyoto Scale of Psychological Development 2001.

To examine test–retest reliability and concurrent validity of the J‐ASQ‐3, data were collected from the Developmental Evaluation Center in the NCCHD and the Nico Children's Clinic in Setagaya. The study protocol was independently approved by the Institutional Review Board of the NCCHD (524, 545, 571, 583, 594, 623, 665, and 671). Participants were parents of children who had been referred to one of these outpatient clinics due to a developmental concern because of a variety of risk factors (e.g. preterm birth, neurological disorder) and parents of children who visited the clinics for regular health check‐ups or for treatment of a minor or self‐limiting illness. Written informed consent was obtained from all participants. In total, parents of 491 children (310 from NCCHD and 181 from Nico) aged 5–66 months were recruited (Fig. 1).

Data were collected between January 2012 and December 2016. After completing the informed consent process, parents were asked to complete the same J‐ASQ‐3 twice: once during the outpatient service and once at home less than or equal to 3 weeks after that. Parents were not informed of the results of the first questionnaire until they had completed the second questionnaire. While the parents were completing the J‐ASQ‐3 at the outpatient clinic, in a different room their child was completing the KSPD and the J‐Denver II, administered by either a psychologist or a speech‐language‐hearing therapist with sufficient training, who was blind to the J‐ASQ‐3 results. Not all the participants could complete this procedure, and the number of the participants whose data were analyzed varied depending on the analyses.

##### Test–retest reliability sample

For test–retest reliability, data were excluded if the J‐ASQ‐3 questionnaires included three or more missing responses in a domain, or if the interval between the two questionnaires was greater than 3 weeks. After this exclusion, data were analyzed for 332 children (177 boys and 155 girls; mean gestational age, 36.9 ± 4.2 weeks; birthweight, 2,609 ± 827 g). Of these children, 165 (49.7%) had been diagnosed with a disorder/condition with the 10th revision of the International Statistical Classification of Diseases and Related Health Problems (ICD‐10) code F, G, or P (mental or behavioral disorder, disease of the nervous system, or condition originating in the perinatal period), which directly increases the risk of developmental delay.

##### Concurrent validity sample

For concurrent validity, data were excluded if the reference test was not planned due to a lack of clinical necessity or could not be administered or scored using standardized methods, or if the parent failed to answer three or more items in one domain of the J‐ASQ‐3 questionnaire. After this exclusion, data for 308 children (191 boys and 117 girls; mean gestational age, 35.9 ± 4.6 weeks; birthweight, 2,415 ± 869 g) were used for comparison with the KSPD (sample K) and data from 309 children (187 boys and 122 girls; mean gestational age, 36.1 ± 4.3 weeks; birthweight, 2,440 ± 865 g) were used for comparison with the J‐Denver II (sample D). The number of children diagnosed with an ICD‐10 code F, G, or P disease was 211 (68.5%) in sample K and 210 (67.1%) in sample D. As the developmental tests were not commonly administered to children who visited the outpatient clinic for a minor illness, these children were often excluded from the concurrent validity analyses, and this increased the proportion of children with ICD‐10 code F, G, or P diagnoses in the concurrent validity analyses. Figure 1 contains more detailed information about the number of participants included in each analysis.

### Measures

#### J‐ASQ‐3

The ASQ‐3 is a screening tool for developmental delay designed for children aged 1–66 months. The tool consists of 21 parent‐rated questionnaires, each of which covers a different age range. Each questionnaire contains 30 items divided into five developmental domains (six items per domain): communication, gross motor skills, fine motor skills, problem solving, and personal–social. For each item, the parent is asked to respond ‘yes’, if their child can do the activity, ‘sometimes’ if their child can sometimes do the activity, and ‘not yet’ if their child cannot do the activity. The responses “yes”, “sometimes,” and “not yet” correspond to a score of 10, 5, and 0, respectively; the total score thus ranges from 0 to 60 for each domain. For each questionnaire, a cut‐off score is determined for each domain. According to the instructions,^1^ a score between 2 SD below the mean and 1 SD below the mean is in the “monitoring zone” for which rescreening is recommended. A score of more than 2 SD below the mean is the referral cut‐off, and indicates need for further assessment. The manual for the original ASQ recommends that a child be considered as screen positive if his/her score falls below the referral cut‐off in any one of the five domains. An alternative deficit criterion of failure in at least two domains has also been used in some previous studies,^13^, ^14^, ^15^ and its validity has been supported. This study used 10 of the ASQ questionnaires (those for 6, 12, 18, 24, 30, 36, 42, 48, 54, and 60 months). To translate the questionnaires into Japanese, the following back‐translation procedure was used. First, a Japanese‐native bilingual speaker translated the questionnaires into Japanese with support from monolingual non‐professional Japanese individuals. Next, an English‐native bilingual speaker translated the Japanese version back into English. After this, another English‐native bilingual compared the back‐translated version with the original version of the questionnaires and evaluated the similarity between them. This series of steps was repeated until the back‐translated version became compatible with the original version. The compatibility between the two versions was also confirmed by Brooks Publishing, leading to their approval of the finalized translation as the Japanese version of the ASQ‐3 questionnaires.

#### KSPD

The KSPD is a standardized developmental test that has been widely used in clinical settings in Japan.^6^ The test provides an overall developmental age and a total developmental quotient, which is calculated as estimated developmental age divided by chronological age. The KSPD also yields a developmental age and a developmental quotient in each of three distinct developmental domains: posture–motor, cognitive–adaptive, and language–social. The posture–motor domain consists of items measuring gross motor skills, such as taking a few steps forward, and climbing stairs using a handrail. The cognitive–adaptive domain consists of items that assess non‐verbal cognitive skills, such as stacking four blocks, and pointing to correct shapes. The language–social domain consists of items that assess verbal cognitive skills, such as recognizing specific words, and repeating a sentence.

For this study, a total developmental quotient of 70 was selected as the cut‐off score to distinguish children with developmental delay from those without. This is at least 2 SD below the mean total developmental quotient of 100, taking account of the SD for each age group of the standardization sample, which ranged from 7 to 12.

#### J‐Denver II

The J‐Denver II was created to screen for developmental delay.^8^ The test consists of 125 items divided into four developmental areas: personal–social, language, fine motor skills, and gross motor skills. During the standardization process, the developer calculated ages at which 25%, 50%, 75%, and 90% of children could perform the task represented by each item. A “caution” is indicated if a child fails to perform a task that 75–90% of same‐aged children can perform, and a “delay” is indicated if a child fails to perform a task that 90% of same‐aged children can perform. Children with one or more delays or two or more cautions are considered as possibly developmentally delayed (screening positive). The J‐Denver II uses all the items from the original Denver II except for three items that do not reflect Japanese culture. The test was re‐standardized using data from 1,819 Japanese children. The criteria used to determine the screening results were the same as those used in the original Denver II. To evaluate preterm children, corrected gestational age was used.

### Statistical analysis

The J‐ASQ‐3 score distribution was explored by calculating the mean and SD for each subscale on each of the 10 J‐ASQ‐3 questionnaires used in this study. For each subscale on each questionnaire, the *t*‐test was used to compare the score on the J‐ASQ‐3 with the score on the original ASQ‐3, which is presented in the ASQ‐3 manual, to investigate the differences between characteristics of the present sample and those of the one used to validate the original ASQ‐3, and a new cut‐off score was determined using the sample mean and SD. For example, if the mean score of a subscale was 15.0 and the SD was 2.5, the cut‐off score (of 2 SD below the mean) is 10.0. The cut‐off scores determined here were used in subsequent analyses to test the concurrent validity of the J‐ASQ‐3.

Test–retest reliability for each subscale of each of the J‐ASQ‐3 questionnaires was calculated using intraclass correlations (ICC) between the scores for the first and second responses to the same questionnaire.^16^ ICC were derived using a mean‐rating (*k* = 2), absolute‐agreement, two‐way mixed‐effects model. Based on Rosner's criteria, ICC >0.75 indicates excellent reliability and that falling between 0.4 and 0.75 indicates fair–good reliability.^17^

Internal consistency of each subscale of each of the J‐ASQ‐3 questionnaires was calculated using Cronbach's alpha. Pearson product‐moment correlation coefficients between the total J‐ASQ‐3 score and the scores for each developmental area were also calculated. According to Tavakol and Dennick, a correlation >0.60 indicates a good level of internal consistency.^18^

Given that the J‐ASQ‐3 is a screening tool for possible developmental delay that requires further assessment, high concurrent validity is indicated when it can accurately identify children classified as being delayed on other screening tools, with high sensitivity and specificity. The KSPD and the J‐Denver II, which can be used to identify children with developmental delay, were used as reference tools. Sensitivity and specificity were calculated by comparing the classification result on the J‐ASQ‐3 with that on the KSPD and the J‐Denver II. Positive and negative predictive values were not calculated because the prevalence of developmental delay in the participants would be higher than the prevalence expected in a community screening setting, and thus these values would not provide relevant information for future studies. Instead, for clinical utility, the likelihood ratio and odds ratio were calculated. Specificity, sensitivity, likelihood ratios, and odds ratios were calculated for the whole sample and separately for each of five different sets of questionnaires (6 and 12 months, 18 and 24 months, 30 and 36 months, 42 and 48 months, and 54 and 60 months). For the J‐ASQ‐3, the original deficit criterion was used, whereby a child was considered delayed if his/her score was below the referral cut‐off in any one of the five domains, and the alternate criterion, whereby the number of domains a child needed to fail to be classified as delayed, was changed from one to two.

It is important to note how missing items in the analyses were dealt with. According to the ASQ‐3 manual, if a domain has one or two missing items, the domain score should be calculated by summing the scores of the remaining items and multiplying the score by 1.2 or 1.5, respectively. If a domain has three or more missing items, it should be excluded from the analyses.^1^ These were the basic guidelines, and different methods were adopted depending on the analysis. When score distribution, new cut‐off scores, and internal consistency were calculated, a method called available item analyses (AIA; data were analyzed with only available items) was used to prevent the values and scores being affected by any inference.^19^ In contrast, test–retest reliability and concurrent validity were derived after values of the missing items were imputed using the mean of the other items in the same domain in accordance with the ASQ‐3 manual. For test–retest reliability, the values were also calculated using AIA because 16% of the data included missing items, and multiple imputation could not be performed due to a lack of variables for determining the conditional distribution of the data.^19^ The number of participants with missing responses in each dataset is shown in Tables S3–S5.

For all statistical analyses, STATA 14.2 (StataCorp LP, College Station, TX, USA) was used.

## Results

### J‐ASQ‐3 score distribution and cut‐off scores

The descriptive statistics for the J‐ASQ‐3 obtained from the participants in the JECS pilot study are presented in Table 1. For almost all subscales and questionnaires, the J‐ASQ‐3 score was significantly different from the score of the original ASQ‐3, obtained from the ASQ‐3 manual. For the questionnaires for younger children, the scores tended to be lower for the J‐ASQ‐3. Specifically, large differences were found on the personal–social and communication subscales. For the questionnaires for older children, however, the scores tended to be higher for the J‐ASQ‐3, although (except for the fine motor skills subscale on the 42 and 48 month questionnaires) the differences were all smaller than 5 points.

Based on the calculated means and SD, cut‐off scores were determined for each subscale of each J‐ASQ‐3 questionnaire (Table 1). For the communication subscale of the 12 and 24 month questionnaires, the gross motor skills subscale of the 12 month questionnaire, and the personal–social subscale of the 6 and 12 month questionnaires, the J‐ASQ‐3 cut‐off score was lower than the original ASQ‐3 cut‐off score by >10 points. In contrast, for the fine motor skills subscale of the 42 and 48 month questionnaires, the J‐ASQ‐3 cut‐off score was higher than the original ASQ‐3 cut‐off score by >10 points.

### Test–retest reliability

Table 2 lists the ICC between the first and second test scores for each subscale of each questionnaire, ranging from 0.62 to 0.97 (mean ICC, 0.84). The magnitude of the correlation was similar across the subscales and the questionnaires, and did not depend on the method used to handle missing data.

**Table 2:** Intraclass correlations between scores on the first and second tests for each subscale and questionnaire (*n* = 368)

Questionnaire (months)	*n*	Communication	Gross motor	Fine motor	Problem solving	Personal–social
ICC for non‐imputed data						
6	29	0.85	0.75	0.72	0.81	0.85
12	12	0.96	0.97	0.87	0.85	0.87
18	36	0.78	0.96	0.81	0.71	0.68
24	22	0.94	0.92	0.84	0.67	0.74
30	43	0.93	0.89	0.65	0.89	0.93
36	48	0.90	0.78	0.72	0.76	0.86
42	22	0.86	0.95	0.88	0.71	0.78
48	17	0.81	0.78	0.83	0.87	0.86
54	19	0.81	0.93	0.90	0.96	0.94
60	33	0.85	0.82	0.91	0.90	0.93
ICC for imputed data						
6	34	0.75	0.75	0.76	0.81	0.87
12	13	0.97	0.97	0.87	0.84	0.90
18	38	0.79	0.95	0.81	0.72	0.68
24	26	0.93	0.92	0.88	0.75	0.76
30	54	0.90	0.91	0.62	0.87	0.91
36	56	0.91	0.81	0.74	0.80	0.88
42	28	0.86	0.94	0.79	0.77	0.79
48	23	0.81	0.76	0.86	0.83	0.87
54	21	0.79	0.85	0.85	0.97	0.94
60	39	0.83	0.80	0.92	0.86	0.92

Numbers of participants were larger for ICC of the imputed datasets because data with missing items were excluded when a dataset was not imputed. ICC, intraclass correlation.

### Internal consistency

Table 3 lists the Cronbach's alpha for each subscale of each J‐ASQ‐3 questionnaire. Cronbach's alpha ranged from 0.45 to 0.89 (mean, 0.69), and was higher for the communication subscales (mean, 0.77) and lower for the personal–social subscales (mean, 0.63). The correlations between subscale scores and total scores are shown in Table S6. For the subscales except for two of them, Pearson product‐moment correlation coefficients were between 0.63 and 0.89. The two exceptions were the communication subscale of the 6 month questionnaire (*r* = 0.53) and the gross motor skills subscale of the 24 month questionnaire (*r* = 0.58).

**Table 3:** Cronbach's alphas for the five subscales in each version of the J‐ASQ‐3

Questionnaire (months)	Communication	Gross motor	Fine motor	Problem solving	Personal–social
6	0.62	0.61	0.73	0.60	0.61
12	0.70	0.87	0.61	0.66	0.74
18	0.72	0.77	0.66	0.71	0.49
24	0.85	0.51	0.45	0.63	0.54
30	0.84	0.57	0.74	0.71	0.65
36	0.82	0.72	0.70	0.70	0.66
42	0.78	0.59	0.66	0.73	0.74
48	0.81	0.69	0.74	0.76	0.60
54	0.89	0.79	0.70	0.74	0.64
60	0.72	0.63	0.81	0.80	0.64

See Table 1 for the numbers of participants assessed with each questionnaire. J‐ASQ‐3, Japanese version of the Ages and Stages Questionnaires, third edition.

### Concurrent validity

The sensitivity and specificity of the J‐ASQ‐3 in comparison with the KSPD and J‐Denver II are given in Table 4. When the KSPD was used to identify children with developmental delay and the original screening criterion of the J‐ASQ‐3 (failure in at least one domain) was used, the sensitivity ranged from 90.9 to 100.0% and the specificity from 43.2 to 63.0%. When the alternative screening criterion of the J‐ASQ‐3 (failure in at least two domains) was used, the sensitivity ranged from 79.0% to 100.0% and the specificity from 68.2 to 93.5%, except for the set of questionnaires for the youngest children (6 and 12 months old), for which it was 54.6%.

**Table 4:** Concurrent validity calculated using the KSPD and the J‐Denver‐II

Questionnaire and sample size for each analysis	No. J‐ASQ‐3 domains screened positive	No. participants screened positive	Sensitivity (%)	Specificity (%)	Correctly classified (%)	Likelihood ratio (+)	Likelihood ratio (−)
KSPD (cut‐off = DQ < 70)							
Total	≥1	203	96.0	48.8	64.3	1.9	0.1
*n* = 308	≥2	145	92.1	74.9	80.5	3.7	0.1
6, 12 months	≥1	31	100.0	45.5	70.7	1.8	0.0
*n* = 41	≥2	25	79.0	54.6	65.9	1.7	0.4
18, 24 months	≥1	39	100.0	43.9	59.7	1.8	0.0
*n* = 57	≥2	25	100.0	78.1	84.2	4.6	0.0
30, 36 months	≥1	51	95.7	46.3	61.0	1.8	0.1
*n* = 77	≥2	38	95.7	70.4	77.9	3.2	0.1
42, 48 months	≥1	45	90.9	43.2	59.1	1.6	0.2
*n* = 66	≥2	34	90.9	68.2	75.8	2.9	0.1
54, 60 months	≥1	37	95.2	63.0	73.1	2.6	0.1
*n* = 67	≥2	23	95.2	93.5	94.0	14.6	0.1
J‐Denver‐II screening positive							
Total	≥1	198	75.6	74.7	75.4	3.0	0.3
*n* = 309	≥2	139	56.3	93.0	64.7	8.0	0.5
6, 12 months	≥1	32	84.2	100.0	86.1		0.2
*n* = 43	≥2	26	68.4	100.0	72.1		0.3
18, 24 months	≥1	40	82.6	85.7	83.3	5.8	0.2
*n* = 60	≥2	23	50.0	100.0	61.7		0.5
30, 36 months	≥1	55	75.8	66.7	73.3	2.3	0.4
*n* = 86	≥2	38	58.1	91.7	67.4	7.0	0.5
42, 48 months	≥1	36	72.1	66.7	70.7	2.2	0.4
*n* = 58	≥2	29	62.8	86.7	69.0	4.7	0.4
54, 60 months	≥1	35	65.3	76.9	67.7	2.8	0.5
*n* = 62	≥2	23	44.9	92.3	54.8	5.8	0.6

DQ <70 in the KSPD and counting at least one delay or two or more cautions in the J‐Denver‐II were used as cut‐off points. The likelihood ratio (+) and/or odds ratio could not be calculated when the sensitivity or specificity was 100.0%. DQ, developmental quotient; J‐ASQ‐3, Japanese version of the Ages and Stages Questionnaires, third edition; J‐Denver‐II, Japanese version of the Denver Developmental Screening Test; KSPD, Kyoto Scale of Psychological Development 2001.

When the J‐Denver II was used to identify children with developmental delay and the original criterion of the J‐ASQ‐3 was used, the sensitivity ranged from 65.3 to 84.2% and the specificity from 66.7 to 100.0%. When the alternative screening criterion of the J‐ASQ‐3 was used, the sensitivity ranged from 44.9 to 68.4% and the specificity ranged from 86.7 to 100.0%.

With regard to the age‐related change in sensitivity and specificity, no specific pattern was found. Different patterns were observed depending on the tool utilized to identify developmental delay and the J‐ASQ‐3 criterion used. Tables S7,S8 list the number of participants who scored below the cut‐off for each of the criteria, and the differences in specificity and sensitivity derived using different ASQ or J‐ASQ‐3 screening criteria (i.e. number of subdomains a child is required to score below to be regarded as screening positive).

## Discussion

The aim of this study was to quantify the psychometric profile of the J‐ASQ‐3, including its score distribution, test–retest reliability, internal consistency, and concurrent validity. Regarding the score distribution, there was a significant difference in scores between the J‐ASQ‐3 and the original ASQ‐3 for almost all subscales on each questionnaire used. The mean scores of the J‐ASQ‐3 subscales tended to be lower than those of the original ASQ‐3 on the questionnaires for younger children, particularly for the personal–social and communication subscales. This is consistent with research comparing median scores on the J‐Denver II and the original Denver II, which showed that Japanese children generally develop slower than US children until approximately 2 years of age.^8^ Compared with US children, Japanese children acquire the following skills >2 months later: removing clothing, speaking a meaningful word, speaking three words, speaking two‐word sentences, pointing at six body parts, naming four pictures correctly, making understandable speech, and walking backwards. In contrast, the mean scores on the J‐ASQ‐3 fine motor skills subscale on the 42 and 48 months questionnaires were higher than those of the original ASQ‐3 by >5 points. A study that compared the pencil grip of Japanese children and English children also found more advanced fine motor skills in Japanese children at preschool age.^20^ Taking into account the consistency in developmental patterns observed with several previous studies, the differences in score distribution between the J‐ASQ‐3 and the original ASQ‐3 found here seem to reflect cultural differences in living environment, rather than a lack of validity. They may reflect opportunities for practicing specific skills in the home or institutional settings, such as the frequency of using pens in daily life. We consider that the cut‐off scores for the J‐ASQ‐3 subscales determined here are applicable to other studies.

The J‐ASQ‐3 subscales had good test–retest reliability in reference to Rosner's criteria:^17^ the values were >0.75 for the gross motor skills and fine motor skills subscales on all questionnaires, and for the other three subscales on almost all questionnaires. It should be noted that the second test was completed less than or equal to 3 weeks after the first test, and at a difference place from the first test. Therefore, taking into account the small age range of each questionnaire, we consider the J‐ASQ‐3 responses to be stable across time and place.

Regarding internal consistency, Cronbach's alpha ranged from 0.45 to 0.89. Except for the gross motor skills subscale on the 24 and 30 month questionnaires, the fine motor skills subscale on the 24 months questionnaire, and the personal–social subscale on the 18 and 24 months questionnaires, the internal consistency was adequate (>0.60).^18^ This is reasonable considering that each of the J‐ASQ‐3 subscales consists of six tasks of different degrees of difficulty, which inevitably decreases the scale's internal consistency. In fact, the internal consistency for the J‐ASQ‐3 subscales found here is similar to that of the original ASQ‐3. Correlations between the subscale scores and the total score ranged from 0.63 to 0.89. This indicates that scores on all J‐ASQ‐3 subscales are sufficiently related to the child's overall development.

This study also examined the concurrent validity of the J‐ASQ‐3. According to these results, sensitivity was high (>90.0%) when the KSPD was used to detect children with developmental delay, regardless of the screening criteria of the J‐ASQ‐3, except for the youngest age range with adoption of the alternative screening criterion. This high sensitivity indicates that the J‐ASQ‐3 can identify most children who require further assessment due to possible delay because the KSPD detects children with developmental delay. Specificity, however, depended on the J‐ASQ‐3 criterion and participant age: it was high (69.2–92.5%) when the alternative criterion for children older than 18 months was used, but moderate (42.1–65.0%) in the other conditions. This indicates that the J‐ASQ‐3 effectively detected children without developmental problems when the alternative criterion for children older than 18 months was used.

Concurrent validity was also acceptable when the J‐Denver‐II was used as the comparison. The English‐language version of the Denver II overidentified children as being delayed;^9^ therefore, a combination of moderate sensitivity and high specificity is desirable when comparing with this scale. When the alternative criterion was used for the J‐ASQ‐3, sensitivity ranged from 45.5 to 71.1% and specificity ranged from 85.7 to 100.0%. Taken together with the results obtained using the KSPD, this suggests that the J‐ASQ‐3 can adequately detect children who are not developmentally delayed.

For the concurrent validity analyses, it is worthwhile to compare the results obtained using the original criterion of the J‐ASQ‐3 with those obtained using the alternative criterion. When the KSPD was used as the reference, the specificity of the J‐ASQ‐3 was much higher with the alternative criterion than with the original criterion, and the sensitivity was similar. In contrast, when the J‐Denver II was used as the reference, the specificity of the J‐ASQ‐3 was much higher with the alternative criterion than with the original criterion, but the sensitivity was lower. As mentioned before, however, for this comparison, moderate sensitivity is more desirable than high sensitivity because of the characteristics of the J‐Denver II, and thus the alternative criterion can still be regarded as better than the original. These results lead us to conclude that the alternative criterion for the J‐ASQ‐3 (failure in at least two domains) worked better than the original criterion (failure in at least one domain) in distinguishing children who needed specialized support from those who did not. This result is compatible with that of the validation study of the Turkish translation of the ASQ‐3,^14^ which also supported the superiority of the alternative criterion, although the validation study of the French translation with preterm birth infants showed no superiority of the alternative criterion over the original criterion,^13^ and that of the original English version with Canadian children showed superiority of the original criterion.^15^ A difference in the testing batteries used for the reference is one likely reason for the inconsistency between the results. According to the manual, validity of the original ASQ‐3 was confirmed using the Bayley‐3. Of the validation studies cited here, Bayley‐3 was used only in the study conducted by Limbos and Joyce,^15^ which supported the superiority of the original criterion. When another testing battery is used as the reference, the alternative criterion produces higher concurrent validity. This means that children may need to be more severely impaired to be regarded as being delayed for the alternative reference batteries than for the Bayley‐3. In support of this, a study that compared the KSPD with the Bayley‐2, an older version of the Bayley‐3, found that the KSPD score was higher than the Bayley‐2 score, suggesting that the children identified as delayed on the KSPD were more severely delayed than those identified on the Bayley‐2.^21^ This supports our explanation of why the alternative criterion for the J‐ASQ‐3 worked better than the original criterion for distinguishing children who needed specialized support from those who did not in the present study.

In the current study, we calculated sensitivity and specificity separately for each of five age groups. Concurrent validity did not seem to be associated with age, which is consistent with the results of the validity study of the original ASQ‐3 using 1.5 SD below the mean as the cut‐off score for the reference test, the Bayley‐3. Our result, however, is inconsistent with that of Schonhaut *et al*., who reported that sensitivity and specificity were higher for the group of older children.^22^ Such a difference might be associated with differences in the reference test and cut‐off score used to identify delayed children. As described in the preceding paragraph, a total developmental quotient of 70 on the KSPD might be a more severe cut‐off than 2 SD below the mean of the Bayley‐3, let alone its 1 SD below the mean, which was used by Schonhaut *et al*. Therefore, when used for young children, it is possible that the ASQ‐3 is not the best tool to identify those with mild developmental delay, but is effective at identifying those with more severe delay.

This study has several strengths. First, the normed sample (the participants of the JECS pilot study) was representative of typical Japanese children. The cut‐off scores for the original ASQ‐3 were established using data from typical US children;^1^ therefore, the J‐ASQ‐3 cut‐off scores established here are similar to those of the original ASQ‐3. Second, we used a community sample to determine the cut‐off scores for the J‐ASQ‐3 subscales and a clinical sample to examine concurrent validity (calculating sensitivity and specificity), following a procedure similar to that used for the original ASQ‐3. This enabled us to effectively estimate the scale validity and its cut‐off scores. Additionally, the use of two different J‐Denver II cut‐off criteria resulted in a more comprehensive estimation of the scale validity.

The study also has several limitations. First, the J‐ASQ‐3 cut‐off scores were calculated using data collected from participants living in a limited range of geographical locations in Japan. The score distribution derived from this study might not be representative of the score distribution from Japanese children in other parts of the country. In fact, the percentages of children born preterm (7.5%) and those born with a low birthweight (12.1%) in the present sample were higher than those for Japanese babies born in 2010 (4.7% and 8.3%), according to Japanese vital statistics reported by Takemoto *et al*.^23^ Therefore, the score distribution should be confirmed in a further study using data from children living in a greater range of locations (such as the JECS main study). Second, the design of the JECS pilot study meant that the respondents for the different J‐ASQ‐3 questionnaires partially overlapped, that is, some mothers completed different questionnaires sequentially in several data collection periods. Therefore, the data (particularly for older ages) might have been influenced by a practice effect that increased the score means and the cut‐off scores of the subscales. Such an effect, however, seems to be negligible given that the concurrent validity of the scale did not substantially differ across the questionnaires. Furthermore, even if cut‐off scores increased, it would not necessarily reduce the appropriateness of the scale as a screening tool, given that the primary purpose of a screening tool is to accurately detect individuals with a problem. Third, for data used for test–retest reliability, 15% of respondents did not answer one or two items in a domain, which may affect the generalizability of the results. Further replication with another dataset might be required to confirm the high test–retest reliability.

In conclusion, we quantified the psychometric profiles of the Japanese translations of 10 ASQ‐3 questionnaires. We demonstrated the validity of the J‐ASQ‐3 and determined new cut‐off scores. Further studies with larger samples from a greater range of locations are necessary to clarify the suitability of this tool for all Japanese children.

## Disclosure

The authors declare no conflict of interest.

## Author contributions

H.Me., H.N., Y.O., K.H. contributed to the conception and design of this study; H.Me., S.A. drafted the manuscript; H.Me. performed the statistical analysis; N.I., K.Ka., S.T., M.T., M.S., S.O., M.O., H.Mi., A.S, K.Ku., M.K. collected data; S.F.N., T.K. gave conceptual advice; K.H. critically reviewed the manuscript and supervised the whole study process. All authors read and approved the final manuscript.

## Supporting information


**Table S1** Demographic information of participants in the JECS pilot study.
**Table S2** Total no. questionnaires completed during the entire pilot study.
**Table S3** No. participants with missing responses for each domain in the samples for the test–retest reliability analysis.
**Table S4** No. participants with missing responses for each domain in the samples for the concurrent validity analyses using the KSPD.
**Table S5** No. participants with missing responses for each domain in the samples for the concurrent validity analyses using the J‐Denver‐II.
**Table S6** Correlations between J‐ASQ‐3 total scores and domain scores for each questionnaire.
**Table S7** Changes in original ASQ/J‐ASQ‐3 sensitivity/specificity depending on screening criteria when using the KSPD.
**Table S8** Changes in original ASQ/J‐ASQ‐3 sensitivity/specificity depending on screening criteria when using the J‐Denver‐II.
