# Supplementary material for: Psychometric profile of the Ages and Stages Questionnaires, Japanese translation
Source: Pediatr Int. 2019 Nov 26;61(11):1086–95. doi: 10.1111/ped.13990 (PMC6899956; doi:10.1111/ped.13990)
Supplement: Supplementary file 1 — Table S1 Demographic information of participants in the JECS pilot study. Table S2 Total no. questionnaires completed during the entire pilot study. Table S3 No. participants with missing responses for each domain in the samples for the test–retest reliability analysis. Table S4 No. participants with missing responses for each domain in the samples for the concurrent validity analyses using the KSPD. Table S5 No. participants with missing responses for each domain in the samples for the concurrent validity analyses using the J‐Denver‐II. Table S6 Correlations between J‐ASQ‐3 total scores and domain scores for each questionnaire. Table S7 Changes in original ASQ/J‐ASQ‐3 sensitivity/specificity depending on screening criteria when using the KSPD. Table S8 Changes in original ASQ/J‐ASQ‐3 sensitivity/specificity depending on screening criteria when using the J‐Denver‐II. [file PED-61-1086-s001.docx]

| Supplementary Table 1 Demographic characteristics of participants in the JECS pilot study. | | | | | |  |
| --- | --- | --- | --- | --- | --- | --- |
| Questionnaire (months) | Number of respondents | Male n (%) | Gestational weeks | | Birth weight | |
|  |  |  | Mean (SD) | Born at < 37 weeks n (%) | Mean (SD) | Born weighing < 2500 g  n (%) |
| Total | 439 | 222 (52.1%) | 38.5 (1.8) | 33 (7.7%) | 2982 (456) | 53 (12.4%) |
| 6 | 48 | 26 (54.2%) | 39.3 (1.2) | 0 (0%) | 3010 (385) | 4 (8.3%) |
| 12 | 155 | 87 (56.1%) | 39.0 (1.2) | 3 (2.0%) | 3029 (400) | 10 (6.5%) |
| 18 | 188 | 104 (55.6%) | 38.8 (1.5) | 8 (4.3%) | 2984 (410) | 22 (11.8%) |
| 24 | 318 | 166 (52.9%) | 38.7 (1.4) | 19 (6.0%) | 3008 (419) | 35 (11.2%) |
| 30 | 326 | 172 (53.6%) | 38.7 (1.5) | 21 (6.5%) | 2999 (424) | 36 (11.2%) |
| 36 | 366 | 186 (52.5%) | 38.6 (1.7) | 25 (7.0%) | 2972 (450) | 46 (13.0%) |
| 42 | 368 | 187 (52.4%) | 38.6 (1.7) | 26 (7.2%) | 2973 (449) | 45 (12.6%) |
| 48 | 354 | 179 (51.9%) | 38.5 (1.7) | 26 (7.5%) | 2974 (447) | 43 (12.4%) |
| 54 | 350 | 176 (51.6%) | 38.5 (1.7) | 26 (7.6%) | 2975 (449) | 43 (12.6%) |
| 60 | 290 | 138 (48.4%) | 38.5 (1.8) | 23 (8.0%) | 2974 (451) | 39 (13.6%) |
| Note: JECS, Japan Environment and Children’s Study. | | | | | | |
|  |  |  |  |  |  |  |
|  |  |  |  |  |  |  |

Supplementary Table 2 Total number of questionnaires that participants completed during the entire pilot study.

| Total number of questionnaires completed | Number of participants |
| --- | --- |
| 0 | 42 |
| 1 | 9 |
| 2 | 7 |
| 3 | 10 |
| 4 | 21 |
| 5 | 25 |
| 6 | 45 |
| 7 | 111 |
| 8 | 83 |
| 9 | 70 |
| 10 | 16 |
| Total | 439 |

Supplementary Table 3 Number of participants with missing responses for each domain in the samples for the test-retest reliability analyses.

| Test sample |  |  |  |  |  |  |  |  |  |  |  |
| --- | --- | --- | --- | --- | --- | --- | --- | --- | --- | --- | --- |
| Questionnaire (months) | Total number of participants | Number of participants with the designated numbers of missing items (one or two) in the domain | | | | | | | | | |
|  |  | Communication | | Gross motor | | Fine motor | | Problem solving | | Personal-social | |
|  |  | One | Two | One | Two | One | Two | One | Two | One | Two |
| Total | 332 | 5 | 1 | 7 | 1 | 6 | 1 | 8 | 0 | 2 | 0 |
| 6 | 34 | 1 | 0 | 2 | 0 | 2 | 0 | 1 | 0 | 0 | 0 |
| 12 | 13 | 0 | 0 | 0 | 0 | 0 | 0 | 0 | 0 | 0 | 0 |
| 18 | 38 | 0 | 0 | 1 | 0 | 0 | 0 | 0 | 0 | 0 | 0 |
| 24 | 26 | 0 | 0 | 0 | 0 | 0 | 0 | 3 | 0 | 1 | 0 |
| 30 | 54 | 1 | 0 | 0 | 1 | 2 | 1 | 1 | 0 | 0 | 0 |
| 36 | 56 | 2 | 0 | 1 | 0 | 0 | 0 | 1 | 1 | 0 | 0 |
| 42 | 28 | 0 | 1 | 2 | 0 | 0 | 0 | 1 | 0 | 0 | 0 |
| 48 | 23 | 0 | 0 | 1 | 0 | 0 | 0 | 0 | 0 | 1 | 0 |
| 54 | 21 | 0 | 0 | 0 | 0 | 1 | 0 | 0 | 0 | 0 | 0 |
| 60 | 39 | 1 | 0 | 0 | 0 | 1 | 0 | 1 | 0 | 0 | 0 |
| Retest sample |  |  |  |  |  |  |  |  |  |  |  |
| Questionnaire (months) | Total number of participants | Number of participants with the designated numbers of missing items (one or two) in the domain | | | | | | | | | |
|  |  | Communication | | Gross motor | | Fine motor | | Problem solving | | Personal-social | |
|  |  | One | Two | One | Two | One | Two | One | Two | One | Two |
| Total | 332 | 5 | 0 | 1 | 0 | 5 | 0 | 4 | 2 | 11 | 0 |
| 6 | 34 | 0 | 0 | 0 | 0 | 0 | 0 | 0 | 0 | 0 | 0 |
| 12 | 13 | 0 | 0 | 0 | 0 | 0 | 0 | 0 | 1 | 0 | 0 |
| 18 | 38 | 0 | 0 | 0 | 0 | 0 | 0 | 0 | 0 | 1 | 0 |
| 24 | 26 | 0 | 0 | 0 | 0 | 0 | 0 | 0 | 0 | 0 | 0 |
| 30 | 54 | 1 | 0 | 0 | 0 | 1 | 0 | 3 | 0 | 1 | 0 |
| 36 | 56 | 3 | 0 | 0 | 0 | 0 | 0 | 0 | 0 | 1 | 0 |
| 42 | 28 | 1 | 0 | 0 | 0 | 1 | 0 | 0 | 1 | 2 | 0 |
| 48 | 23 | 0 | 0 | 1 | 0 | 1 | 0 | 0 | 0 | 4 | 0 |
| 54 | 21 | 0 | 0 | 0 | 0 | 1 | 0 | 1 | 0 | 0 | 0 |
| 60 | 39 | 0 | 0 | 0 | 0 | 1 | 0 | 0 | 0 | 2 | 0 |

Supplementary Table 4 Number of participants with missing responses for each domain in the samples for the concurrent validity analyses using the KSPD.

| Questionnaire (months) | Total number of participants | Number of participants with the designated numbers of missing items (one or two) in the domain | | | | | | | | | |
| --- | --- | --- | --- | --- | --- | --- | --- | --- | --- | --- | --- |
|  |  | Communication | | Gross motor | | Fine motor | | Problem solving | | Personal-social | |
|  |  | One | Two | One | Two | One | Two | One | Two | One | Two |
| Total | 308 | 4 | 2 | 10 | 1 | 5 | 1 | 8 | 1 | 4 | 0 |
| 6 | 20 | 1 | 1 | 2 | 0 | 0 | 0 | 0 | 0 | 0 | 0 |
| 12 | 21 | 0 | 0 | 0 | 0 | 0 | 0 | 2 | 0 | 0 | 0 |
| 18 | 27 | 0 | 0 | 1 | 0 | 0 | 1 | 0 | 0 | 0 | 0 |
| 24 | 30 | 0 | 0 | 0 | 0 | 0 | 0 | 3 | 0 | 1 | 0 |
| 30 | 29 | 1 | 0 | 0 | 1 | 1 | 0 | 0 | 0 | 0 | 0 |
| 36 | 48 | 2 | 0 | 1 | 0 | 1 | 0 | 1 | 1 | 0 | 0 |
| 42 | 41 | 0 | 1 | 3 | 0 | 0 | 0 | 1 | 0 | 0 | 0 |
| 48 | 25 | 0 | 0 | 3 | 0 | 0 | 0 | 0 | 0 | 3 | 0 |
| 54 | 30 | 0 | 0 | 0 | 0 | 1 | 0 | 0 | 0 | 0 | 0 |
| 60 | 37 | 0 | 0 | 0 | 0 | 2 | 0 | 1 | 0 | 0 | 0 |
| KSPD, Kyoto Scale of Psychological Development 2001. | | | | |  |  |  |  |  |  |  |

Supplementary Table 5 Number of participants with missing responses for each domain in the samples for the concurrent validity analyses using the J-Denver-II.

| Questionnaire (months) | Total number of participants | Number of participants with the designated numbers of missing items (one or two) in the domain | | | | | | | | | |
| --- | --- | --- | --- | --- | --- | --- | --- | --- | --- | --- | --- |
|  |  | Communication | | Gross motor | | Fine motor | | Problem solving | | Personal-social | |
|  |  | One | Two | One | Two | One | Two | One | Two | One | Two |
| Total | 309 | 4 | 2 | 9 | 1 | 5 | 1 | 9 | 0 | 3 | 1 |
| 6 | 19 | 1 | 1 | 2 | 0 | 0 | 0 | 0 | 0 | 0 | 0 |
| 12 | 24 | 0 | 0 | 0 | 0 | 0 | 0 | 2 | 0 | 0 | 0 |
| 18 | 31 | 0 | 0 | 1 | 0 | 0 | 1 | 0 | 0 | 0 | 0 |
| 24 | 29 | 0 | 0 | 0 | 0 | 0 | 0 | 4 | 0 | 1 | 0 |
| 30 | 33 | 1 | 0 | 0 | 1 | 1 | 0 | 0 | 0 | 0 | 0 |
| 36 | 53 | 2 | 0 | 1 | 0 | 2 | 0 | 1 | 0 | 0 | 0 |
| 42 | 39 | 0 | 1 | 3 | 0 | 0 | 0 | 1 | 0 | 0 | 0 |
| 48 | 19 | 0 | 0 | 2 | 0 | 0 | 0 | 0 | 0 | 2 | 1 |
| 54 | 29 | 0 | 0 | 0 | 0 | 1 | 0 | 0 | 0 | 0 | 0 |
| 60 | 33 | 0 | 0 | 0 | 0 | 1 | 0 | 1 | 0 | 0 | 0 |
| J-Denver-II, Japanese version of the Denver Developmental Screening Test. | | | | | |  |  |  |  |  |  |

| Supplementary Table 6 Correlations between J-ASQ-3 total scores and domain scores for each questionnaire. | | | | | | |
| --- | --- | --- | --- | --- | --- | --- |
| Questionnaire (months) | n | Communication | Gross motor | Fine motor | Problem solving | Personal-social |
| 6 | 46 | 0.53 | 0.69 | 0.87 | 0.66 | 0.76 |
| 12 | 138 | 0.75 | 0.69 | 0.77 | 0.83 | 0.80 |
| 18 | 173 | 0.73 | 0.63 | 0.73 | 0.80 | 0.75 |
| 24 | 301 | 0.79 | 0.58 | 0.73 | 0.80 | 0.79 |
| 30 | 294 | 0.85 | 0.64 | 0.75 | 0.86 | 0.80 |
| 36 | 263 | 0.87 | 0.71 | 0.84 | 0.86 | 0.84 |
| 42 | 339 | 0.89 | 0.68 | 0.81 | 0.85 | 0.85 |
| 48 | 308 | 0.85 | 0.78 | 0.84 | 0.86 | 0.83 |
| 54 | 306 | 0.86 | 0.81 | 0.82 | 0.84 | 0.84 |
| 60 | 190 | 0.79 | 0.82 | 0.83 | 0.86 | 0.81 |
| J-ASQ-3, Japanese version of the Ages and Stages Questionnaires, third edition. | | | | |  |  |

| Supplementary Table 7 Changes in original ASQ/J-ASQ-3 sensitivity/specificity depending on screening criteria when using the KSPD. | | | | | | | | | | | | |  |  | |
| --- | --- | --- | --- | --- | --- | --- | --- | --- | --- | --- | --- | --- | --- | --- | --- |
| Questionnaire and sample size for each analysis | Number of domains screened positive | KSPD cut-off = DQ < 70 | | | | | | | | | | | | |  |
|  |  | Original ASQ-3 | | | | | | J-ASQ-3 | | | | | | |  |
|  |  | Number of participants screened positive | Sensitivity | Specificity | Correctly classified | LR+ | LR- | Number of participants screened positive | Sensitivity | Specificity | Correctly classified | LR+ | LR- | |  |
| Total n = 308 | ≥ 1 | 203 | 97.0% | 49.3% | 64.9% | 1.9 | 0.1 | 203 | 96.0% | 48.8% | 64.3% | 1.9 | 0.1 | |  |
|  | ≥ 2 | 154 | 92.1% | 70.5% | 77.6% | 3.1 | 0.1 | 145 | 92.1% | 74.9% | 80.5% | 3.7 | 0.1 | |  |
|  | ≥ 3 | 110 | 77.2% | 84.5% | 82.1% | 5.0 | 0.3 | 99 | 76.2% | 89.4% | 85.1% | 7.2 | 0.3 | |  |
|  | ≥ 4 | 75 | 63.4% | 94.7% | 84.4% | 11.9 | 0.4 | 67 | 57.4% | 95.7% | 83.1% | 13.2 | 0.4 | |  |
|  | ≥ 5 | 40 | 37.6% | 99.0% | 78.9% | 38.9 | 0.6 | 41 | 36.6% | 98.1% | 77.9% | 19.0 | 0.6 | |  |
| 6, 12 months n = 41 | ≥ 1 | 35 | 100.0% | 27.3% | 61.0% | 1.4 | 0.0 | 31 | 100.0% | 45.5% | 70.7% | 1.8 | 0.0 | |  |
|  | ≥ 2 | 32 | 94.7% | 36.4% | 63.4% | 1.5 | 0.1 | 25 | 79.0% | 54.6% | 65.9% | 1.7 | 0.4 | |  |
|  | ≥ 3 | 28 | 89.5% | 50.0% | 68.3% | 1.8 | 0.2 | 15 | 68.4% | 90.9% | 80.5% | 7.5 | 0.3 | |  |
|  | ≥ 4 | 18 | 79.0% | 86.4% | 82.9% | 5.8 | 0.2 | 7 | 36.8% | 100.0% | 70.7% | *1 | 0.6 | |  |
|  | ≥ 5 | 9 | 42.1% | 95.5% | 70.7% | 9.3 | 0.6 | 1 | 5.3% | 100.0% | 56.1% | *1 | 0.9 | |  |
|  |  |  |  |  |  |  |  |  |  |  |  |  |  | |  |
| 18, 24 months n = 57 | ≥ 1 | 44 | 100.0% | 31.7% | 50.9% | 1.5 | 0.0 | 39 | 100.0% | 43.9% | 59.7% | 1.8 | 0.0 | |  |
|  | ≥ 2 | 32 | 100.0% | 61.0% | 71.9% | 2.6 | 0.0 | 25 | 100.0% | 78.1% | 84.2% | 4.6 | 0.0 | |  |
|  | ≥ 3 | 21 | 87.5% | 82.9% | 84.2% | 5.1 | 0.2 | 16 | 81.3% | 92.7% | 89.5% | 11.1 | 0.2 | |  |
|  | ≥ 4 | 14 | 75.0% | 95.1% | 89.5% | 15.4 | 0.3 | 8 | 50.0% | 100.0% | 86.0% | *1 | 0.5 | |  |
|  | ≥ 5 | 6 | 37.5% | 100.0% | 82.5% | *1 | 0.6 | 5 | 31.3% | 100.0% | 80.7% | *1 | 0.7 | |  |
| 30, 36 months n = 77 | ≥ 1 | 49 | 95.7% | 50.0% | 63.6% | 1.9 | 0.1 | 51 | 95.7% | 46.3% | 61.0% | 1.8 | 0.1 | |  |
|  | ≥ 2 | 40 | 95.7% | 66.7% | 75.3% | 2.9 | 0.1 | 38 | 95.7% | 70.4% | 77.9% | 3.2 | 0.1 | |  |
|  | ≥ 3 | 30 | 91.3% | 83.3% | 85.7% | 5.5 | 0.1 | 26 | 78.3% | 85.2% | 83.1% | 5.3 | 0.3 | |  |
|  | ≥ 4 | 19 | 69.6% | 94.4% | 87.0% | 12.5 | 0.3 | 20 | 73.9% | 94.4% | 88.3% | 13.3 | 0.3 | |  |
|  | ≥ 5 | 9 | 39.1% | 100.0% | 81.8% | *1 | 0.6 | 12 | 47.8% | 98.2% | 83.1% | 25.8 | 0.5 | |  |
| 42, 48 months n = 66 | ≥ 1 | 39 | 90.9% | 56.8% | 68.2% | 2.1 | 0.2 | 45 | 90.9% | 43.2% | 59.1% | 1.6 | 0.2 | |  |
|  | ≥ 2 | 27 | 90.9% | 84.1% | 86.4% | 5.7 | 0.1 | 34 | 90.9% | 68.2% | 75.8% | 2.9 | 0.1 | |  |
|  | ≥ 3 | 19 | 63.6% | 88.6% | 80.3% | 5.6 | 0.4 | 25 | 77.3% | 81.8% | 80.3% | 4.3 | 0.3 | |  |
|  | ≥ 4 | 12 | 40.9% | 93.2% | 75.8% | 6.0 | 0.6 | 17 | 54.6% | 88.6% | 77.3% | 4.8 | 0.5 | |  |
|  | ≥ 5 | 9 | 36.4% | 97.7% | 77.3% | 16.0 | 0.7 | 13 | 45.5% | 93.2% | 77.3% | 6.7 | 0.6 | |  |
| 54, 60 months n = 67 | ≥ 1 | 36 | 100.0% | 67.4% | 77.6% | 3.1 | 0.0 | 37 | 95.2% | 63.0% | 73.1% | 2.6 | 0.1 | |  |
|  | ≥ 2 | 23 | 81.0% | 87.0% | 85.1% | 6.2 | 0.2 | 23 | 95.2% | 93.5% | 94.0% | 14.6 | 0.1 | |  |
|  | ≥ 3 | 12 | *2 | *2 | *2 | *2 | *2 | 17 | 76.2% | 97.8% | 91.0% | 35.0 | 0.2 | |  |
|  | ≥ 4 | 12 | 57.1% | 100.0% | 86.6% | *1 | 0.4 | 15 | 66.7% | 97.8% | 88.1% | 30.7 | 0.3 | |  |
|  | ≥ 5 | 7 | 33.3% | 100.0% | 79.1% | *1 | 0.7 | 10 | 47.6% | 100.0% | 83.6% | *1 | 0.5 | |  |
| Note: This table shows how sensitivity and specificity change when a positive screening requires a child to score below the cutoff in different numbers of domains. Original values were used for analyses with the original ASQ-3, while newly established values were used for analyses with the J-ASQ-3. It should be noted that in the guidelines of the ASQ-3, a child is regarded as screening positive if they score below the cutoff points for any one of the five domains (≥ 1).  *1 LR(+) could not be calculated because all the participants with DQ below 70 were categorized as screened positive.  *2 None of the participants were categorized as screen positive under this condition. J-ASQ-3, Japanese version of the Ages and Stages Questionnaires, third edition; KSPD, Kyoto Scale of Psychological Development 2001; DQ, Developmental Quotient; LR, likelihood ratio. | | | | | | | | | | | | | | |  |
|  |  |  |  |  |  |  |  |  |  |  |  |  |  |  |  |

| Supplementary Table 8 Changes in original ASQ/J-ASQ-3 sensitivity/specificity depending on screening criteria when using the J-Denver-II. | | | | | | | | | | | | |  |
| --- | --- | --- | --- | --- | --- | --- | --- | --- | --- | --- | --- | --- | --- |
| Questionnaire and sample size for each analysis | Number of domains screened positive | J-Denver-II screening positive | | | | | | | | | | | |
|  |  | Original ASQ-3 | | | | | | J-ASQ-3 | | | | | |
|  |  | Number of participants screened positive | Sensitivity | Specificity | Correctly classified | LR+ | LR- | Number of participants screened positive | Sensitivity | Specificity | Correctly classified | LR+ | LR- |
| Total n = 309 | ≥ 1 | 196 | 74.4% | 73.2% | 74.1% | 2.8 | 0.4 | 198 | 75.6% | 74.7% | 75.4% | 3.0 | 0.3 |
|  | ≥ 2 | 149 | 60.1% | 91.6% | 67.3% | 7.1 | 0.4 | 139 | 56.3% | 93.0% | 64.7% | 8.0 | 0.5 |
|  | ≥ 3 | 104 | 43.3% | 98.6% | 56.0% | 30.7 | 0.6 | 91 | 37.4% | 97.2% | 51.1% | 13.3 | 0.6 |
|  | ≥ 4 | 70 | 29.4% | 100.0% | 45.6% | *1 | 0.7 | 63 | 26.1% | 98.6% | 42.7% | 18.5 | 0.8 |
|  | ≥ 5 | 37 | 15.6% | 100.0% | 35.0% | *1 | 0.8 | 36 | 15.1% | 100.0% | 34.6% | *1 | 0.8 |
| 6, 12 months n = 43 | ≥ 1 | 37 | 94.7% | 80.0% | 93.0% | 4.7 | 0.1 | 32 | 84.2% | 100.0% | 86.1% | *1 | 0.2 |
|  | ≥ 2 | 33 | 84.2% | 80.0% | 83.7% | 4.2 | 0.2 | 26 | 68.4% | 100.0% | 72.1% | *1 | 0.3 |
|  | ≥ 3 | 29 | 76.3% | 100.0% | 79.1% | *1 | 0.2 | 15 | 39.5% | 100.0% | 46.5% | *1 | 0.6 |
|  | ≥ 4 | 19 | 50.0% | 100.0% | 55.8% | *1 | 0.5 | 7 | 2.6% | 100.0% | 14.0% | *1 | 1.0 |
|  | ≥ 5 | 10 | 26.3% | 100.0% | 34.9% | *1 | 0.7 | 1 | 0.0% | 100.0% | 11.6% | *1 | 1.0 |
|  |  |  |  |  |  |  |  |  |  |  |  |  |  |
| 18, 24 months n = 60 | ≥ 1 | 45 | 87.0% | 64.3% | 81.7% | 2.4 | 0.2 | 40 | 82.6% | 85.7% | 83.3% | 5.8 | 0.2 |
|  | ≥ 2 | 31 | 65.2% | 92.9% | 71.7% | 9.1 | 0.4 | 23 | 50.0% | 100.0% | 61.7% | *1 | 0.5 |
|  | ≥ 3 | 21 | 45.7% | 100.0% | 58.3% | *1 | 0.5 | 16 | 34.8% | 100.0% | 50.0% | *1 | 0.7 |
|  | ≥ 4 | 14 | 30.4% | 100.0% | 46.7% | *1 | 0.7 | 8 | 17.4% | 100.0% | 36.7% | *1 | 0.8 |
|  | ≥ 5 | 6 | 13.0% | 100.0% | 33.3% | *1 | 0.9 | 5 | 10.9% | 100.0% | 31.7% | *1 | 0.9 |
| 30, 36 months n = 86 | ≥ 1 | 51 | 69.4% | 66.7% | 68.6% | 2.1 | 0.5 | 55 | 75.8% | 66.7% | 73.3% | 2.3 | 0.4 |
|  | ≥ 2 | 40 | 62.9% | 95.8% | 72.1% | 15.1 | 0.4 | 38 | 58.1% | 91.7% | 67.4% | 7.0 | 0.5 |
|  | ≥ 3 | 28 | 45.2% | 100.0% | 60.5% | *1 | 0.5 | 24 | 38.7% | 100.0% | 55.8% | *1 | 0.6 |
|  | ≥ 4 | 16 | 25.8% | 100.0% | 46.5% | *1 | 0.7 | 19 | 30.7% | 100.0% | 50.0% | *1 | 0.7 |
|  | ≥ 5 | 7 | 11.3% | 100.0% | 36.1% | *1 | 0.9 | 10 | 16.1% | 100.0% | 39.5% | *1 | 0.8 |
| 42, 48 months n = 58 | ≥ 1 | 30 | 65.1% | 86.7% | 70.7% | 4.9 | 0.4 | 36 | 72.1% | 66.7% | 70.7% | 2.2 | 0.4 |
|  | ≥ 2 | 23 | 48.8% | 86.7% | 58.6% | 3.7 | 0.6 | 29 | 62.8% | 86.7% | 69.0% | 4.7 | 0.4 |
|  | ≥ 3 | 15 | 32.6% | 93.3% | 48.3% | 4.9 | 0.7 | 19 | 39.5% | 86.7% | 51.7% | 3.0 | 0.7 |
|  | ≥ 4 | 10 | 23.3% | 100.0% | 43.1% | *1 | 0.8 | 15 | 32.6% | 93.3% | 48.3% | 4.9 | 0.7 |
|  | ≥ 5 | 7 | 16.3% | 100.0% | 37.9% | *1 | 0.8 | 11 | 25.6% | 100.0% | 44.8% | *1 | 0.7 |
| 54, 60 months n = 62 | ≥ 1 | 33 | 61.2% | 76.9% | 64.5% | 2.7 | 0.5 | 35 | 65.3% | 76.9% | 67.7% | 2.8 | 0.5 |
|  | ≥ 2 | 22 | 42.9% | 92.3% | 53.2% | 5.6 | 0.6 | 23 | 44.9% | 92.3% | 54.8% | 5.8 | 0.6 |
|  | ≥ 3 | 11 | *2 | *2 | *2 | *2 | *2 | 17 | 34.7% | 100.0% | 48.4% | *1 | 0.7 |
|  | ≥ 4 | 11 | 22.5% | 100.0% | 38.7% | *1 | 0.8 | 14 | 28.6% | 100.0% | 43.6% | *1 | 0.7 |
|  | ≥ 5 | 7 | 14.3% | 100.0% | 32.3% | *1 | 0.9 | 9 | 18.4% | 100.0% | 35.5% | *1 | 0.8 |
| Note: This table shows how sensitivity and specificity change when a positive screening requires a child to score below the cutoff in different numbers of domains. At least one “delay” or two or more “cautions” were used as the J-Denver-II screening criterion. With regard to the cutoff scores for each domain, original values were used for analyses with the original ASQ-3, while newly established values were used for analyses with the J-ASQ-3. It should be noted that in the guidelines of the ASQ-3, a child is regarded as screening positive if they score below the cutoff points for any one of the five domains (≥ 1).  *1 LR(+) could not be calculated because all the participants with J-Denver-II screened positive were categorized as screened positive.  *2 None of the participants were categorized as screen positive under this condition. J-ASQ-3, Japanese version of the Ages and Stages Questionnaires, third edition; J-Denver-II, Japanese version of the Denver Developmental Screening Test; LR, likelihood ratio. | | | | | | | | | | | | | |
|  |  |  |  |  |  |  |  |  |  |  |  |  |  |
